# The Impact of a Formalized Fertility Preservation Program on Access to Care and Sperm Cryopreservation Among Transgender and Nonbinary Patients Assigned Male at Birth

**DOI:** 10.3390/jcm14124203

**Published:** 2025-06-13

**Authors:** Daniel R. Greenberg, Faraz N. Longi, Sarah C. Cromack, Kristin N. Smith, Valerie G. Brown, Sarah E. Bazzetta, Kara N. Goldman, Robert E. Brannigan, Joshua A. Halpern

**Affiliations:** 1 Department of Urology, Northwestern University Feinberg School of Medicine, Chicago, IL 60611, USA; faraz.longi@northwestern.edu (F.N.L.); valerie.gillis@nm.org (V.G.B.); r-brannigan@northwestern.edu (R.E.B.); joshua.halpern@northwestern.edu (J.A.H.); 2 Department of Obstetrics and Gynecology, Northwestern University Feinberg School of Medicine, Chicago, IL 60611, USA; sarah.capelouto@nm.org (S.C.C.); ksmith@nm.org (K.N.S.); sarah.bazzetta@northwestern.edu (S.E.B.); kara.goldman@northwestern.edu (K.N.G.)

**Keywords:** fertility preservation, sperm cryopreservation, gender affirmation, transgender

## Abstract

**Objectives:** This study aimed to evaluate the implementation of a formalized fertility preservation (FP) program for transgender and nonbinary patients assigned male at birth (TGNB-AMAB) at our institution. **Methods**: We reviewed TGNB-AMAB patients who were referred to the FP program at our academic institution between 2016 and September 2023. We compared the number of referrals and the percentage of patients who underwent FP per year. Clinical and demographic information including age at referral, time from referral to banking, semen parameters, and serum hormone values were evaluated. **Results**: In total, 154 TGNB-AMAB patients were referred to the FP program since 2016; 131 (85.1%) met with a reproductive urologist or advanced practice provider for FP consultation; and 124 (94.7%) completed sperm cryopreservation. The number of annual referrals significantly increased over time (*p* = 0.001). The average age (±standard deviation) at referral was 20.5 ± 5.7 years. The median time from referral to sperm cryopreservation was 14 days. The average semen parameters among all the patients were volume 2.7 ± 1.7 mL, sperm concentration 36.0 ± 31.6 M/mL, sperm motility 56.8 ± 19.0%, and sperm morphology 4.7 ± 2.9%. There was no significant difference in semen parameters between TGNB-AMAB patients previously on gender-affirming hormonal therapy prior to banking and those not on prior hormonal treatment (*p* > 0.05). **Conclusions**: Our fertility preservation program significantly increased the number of TGNB-AMAB patients who received consultation and underwent sperm cryopreservation. The institution of a formalized FP program can be used to increase access for TGNB-AMAB patients who desire future fertility.

## 1. Introduction

In the United States, there are approximately 1.4 million transgender individuals—people whose gender identity differs from their biological sex assigned at birth [1]. The physical transition for gender affirmation for transgender and nonbinary individuals assigned male at birth (TGNB-AMAB) may involve hormonal therapy (i.e., anti-androgens, estrogen) and surgery (i.e., orchiectomy, penectomy), both of which can severely decrease or eliminate fertility potential [2,3]. Professional organizations including the World Professional Association for Transgender Health (WPATH), the American Society for Reproductive Medicine, and the Endocrine Society recommend counseling and offering fertility preservation (FP) prior to undertaking gender affirmation hormone therapy (GAHT) [4,5,6]. However, the data show that few TGNB-AMAB patients undergo FP prior to starting GAHT or gender-affirming surgery despite a desire for biological parenthood and a high rate of decisional regret when not initially pursued [7,8].

Sperm cryopreservation remains the gold standard for FP among TGNB-AMAB patients [9]. Stored samples may be used for future assisted reproductive technologies (ARTs) including intrauterine insemination or in vitro fertilization with intracytoplasmic sperm injection if necessary [10]. Patients who have started GAHT prior to sperm cryopreservation often require the cessation of GAHT to improve semen parameters given the downregulatory effects of estrogen and anti-androgens on the hypothalamic–pituitary–gonadal axis [11].

Currently, there are no established guidelines for the duration of hormone cessation, and the results from small, retrospective studies show that changes in semen parameters may be irreversible [12]. Additionally, this interruption of or delay in hormonal therapy is associated with worsening gender dysphoria and is a contributing factor in the low adoption of FP in this population [13,14]. Few prior studies have evaluated semen parameters for transgender women on active GAHT; however, among the available data, the rates of azoospermia are 42–50% [15,16]. Therefore, it is critically important that TGNB-AMAB patients receive appropriate counseling and referral for FP early in the transition process.

As part of the Gender Pathways Program (GPP) at Northwestern University—a multidisciplinary care model designed to meet the medical and surgical needs of TGNB patients—we developed a formalized referral process for the fertility preservation of sperm, oocytes, and embryos. We aimed to evaluate the effect of this formalized FP program on sperm cryopreservation since its inception in 2016. We hypothesized that referrals increased over time and that the rates of sperm cryopreservation improved with a formally instituted referral program.

## 2. Materials and Methods

### 2.1. Fertility Preservation Program Development and Workflow

The FP referral program for TGNB patients was established in 2016 as part of our institution’s development of the GPP. The FP program was nested within the already-existing Oncofertility program at Northwestern University, which was previously established in 2009 [17]. The Oncofertility program focused on FP for newly diagnosed adolescent and young adult cancer patients and therefore was well adapted to provide FP for any patients requiring urgent gonadotoxic therapy.

The GPP combines a multidisciplinary team of specialists to offer a range of surgical, medical, and mental health services for transgender and gender-diverse patients. Upon intake to the GPP, fertility preservation is discussed, and a referral is placed by direct electronic medical record messaging, phone call, or secure email. The Fertility Preservation Navigator will call the patient or family directly to assess patient goals, including desire for FP. If desired, TGNB-AMAB patients are referred to the Department of Urology to discuss sperm cryopreservation and banking with a reproductive urologist (RU) or advanced practice provider (APP) trained in reproductive urology.

Patients were excluded from the analysis if determined to be pre-pubertal at initial evaluation as they would be unable to undergo sperm cryopreservation and were referred for other preservation services through a pediatric physician. All patient care was performed in accordance with the WPATH guidelines on the Standards of Care for the Health of Transgender and Gender Diverse People [4].

### 2.2. Study Variables and Outcomes of Interest

Demographic and clinical data including age at referral, year of referral, and days from referral to sperm banking were evaluated for all the TGNB-AMAB patients in our program since its inception from 2016 to 2023. Sperm cryopreservation variables including number of times banking and vials of semen collected per banking were also collected. Semen parameters including ejaculate volume, sperm concentration, sperm motility, sperm morphology, and total motile sperm count (TMSC) were analyzed. All the semen processing and semen analyses were performed within our healthcare system by an appropriately credentialed member of the IVF and Andrology Laboratory Staff and in accordance with the 2010 *WHO Laboratory Manual for the Examination and Processing of Human Semen* (5th Edition) [18]. Hormonal values including morning (before 11AM) serum testosterone (T) and follicle-stimulating hormone (FSH) were included if obtained within one year of referral. Lastly, prior history of GAHT, time from referral to sperm banking, and time from referral to the initiation of GAHT were recorded.

The primary outcomes of interest were the rate of completing FP referral consultation with an RU/APP and the rate of undergoing sperm cryopreservation over time. The secondary outcomes included bulk semen parameters and serum hormonal values in this population.

Patient informed consent was waived for this study given the retrospective study design and use of deidentified patient data. This study received institutional review board approval from Northwestern University on 22 September 2021 (IRB: STU00215630).

### 2.3. Statistical Analysis

Data were analyzed using Stata SE version 18 (StataCorp LLC, College Station, TX, USA). Descriptive statistics were utilized to characterize the population of TGNB-AMAB patients who were part of our FP referral program. We compared baseline demographics, clinical characteristics, and variables between pediatric/adolescent (<18 years old at the time of referral) and adult (≥18 years old at the time of referral) patients in our cohort. Differences between the cohorts were assessed via Welch’s unpaired Student’s *t*-test and chi-squared tests, as appropriate. Linear regression analysis was used to determine the association between the year of referral and the number of patients referred to the FP program, and the Cochran–Armitage test for trend was used to determine the association between the percentage of TGNB-AMAB patients completing their FP referral and those providing a sample for cryopreservation over time. All tests of significance were two-sided, and statistical significance was determined at a level of *p* < 0.05.

## 3. Results

**Figure 1:**
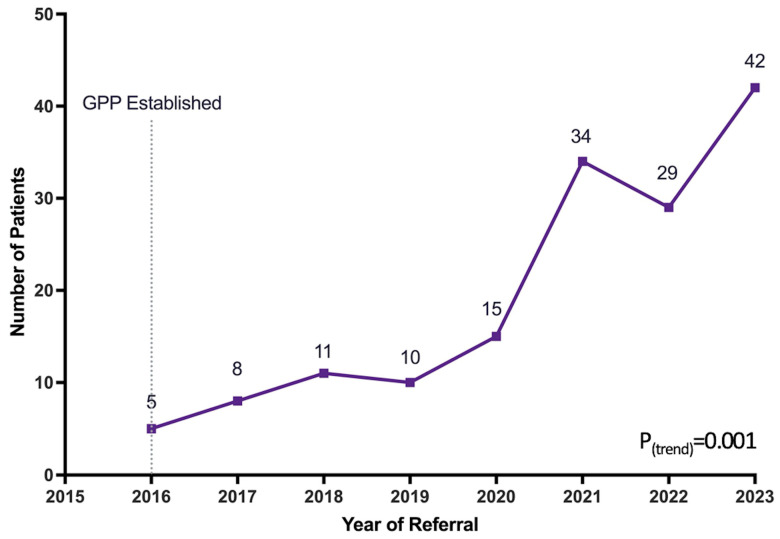
Number of patients referred to the GPP per year.

In total, 154 TGNB-AMAB patients were referred to the FP program between 2016 and 2023. The number of patients referred per year significantly increased over time, starting with 5 patients in 2016 and increasing to 42 patients in 2023 (Figure 1, *p* = 0.001). Despite the increasing number of patients referred per year, the percentage of patients who completed the referral process (underwent consultation with a reproductive urologist or APP) and the percentage of patients who underwent sperm cryopreservation did not change over time. These ranged from 76.2 to 100.0% (*p* = 0.22 for trend over time) and 71.4–100.0% (*p* = 0.24 for trend over time), respectively (Table 1).

The average age of the TGNB-AMAB patients referred to the FP program was 20.5 ± 5.7 years. Overall, 23.4% (n = 36) of patients were on GAHT prior to FP referral, with a significantly greater percentage of adult (≥18 years of age) patients on prior GAHT than pediatric/adolescent patients (31.3% vs. 14.1%, *p* = 0.01). The median age of all patients with prior GAHT at the time of sperm banking was 20.7 years (interquartile range [IQR] 17.3–24.0 years).

Among patients who underwent sperm cryopreservation, the median time from initial referral to sperm banking was two weeks (14 days, IQR 9–29 days). Patients banked sperm an average of 2.1 ± 1.5 times and averaged 4.4 ± 3.1 vials per bank. The average semen parameters among all the TGNB-AMAB patients were as follows: volume 2.7 ± 1.7 mL, sperm concentration 36.0 ± 31.6 M/mL, sperm motility 56.8 ± 19.0%, and sperm morphology 4.7 ± 2.9% (Table 2). Adult patients had a significantly greater ejaculate volume (3.4 ± 1.7 mL vs. 1.9 ± 1.1 mL, *p* < 0.001), percent normal morphology (5.2 ± 2.7% vs. 4.0 ± 2.9%, *p* = 0.03), and TMSC (80.9 ± 60.7 M vs. 36.4 ± 41.1 M, *p* < 0.001) than pediatric/adolescent patients. However, there was no difference in serum testosterone or follicle-stimulating hormone (FSH) between age cohorts. There was also no difference in bulk semen parameters between TGNB-AMAB patients with and without prior utilization of GAHT (Table 3, *p* > 0.05).

Lastly, among patients wishing to pursue GAHT, the median time to starting hormonal therapy after their last banking procedure was approximately two weeks (androgen receptor blocker = 17 days, IQR 6–52 days; estradiol = 13 days, IQR 3–43 days). The overall median time from the initial referral to the initiation of any form of GAHT was one month.

## 4. Discussion

Our institution established the GPP in 2016 to provide multidisciplinary care for TGNB patients with a focus on reproductive health and fertility preservation. Following its inception, we found a significant increase in the number of FP referrals per year for our TGNB-AMAB patients. The implementation of this formalized PF program allowed our institution to maintain a high rate of sperm cryopreservation among patients who completed the referral process. We also demonstrated an expeditious referral process with a time from referral to sperm banking of only two weeks and a similarly short delay from referral to the initiation of GAHT of approximately one month. Lastly, we found no difference in semen parameters between patients with or without prior GAHT. Our study demonstrates that a formalized FP program can adequately address the reproductive needs of TGNB-AMAB patients planning to undergo gender affirmation.

Our FP program saw an increase in the number of annual referrals from 5 patients in 2016 to 42 patients in 2023. In the state of Illinois, transgender FP became covered by insurance under H.B. 2617 as medical fertility preservation on 1 January 2019, which amended the Illinois insurance code to “provide that a policy of accident or health insurance shall provide coverage for medically necessary expenses for standard fertility preservation services when a necessary medical treatment may directly or indirectly cause iatrogenic infertility to an enrollee” [19]. While this legislation likely helped increase referrals, the number of referrals per year had already doubled prior to its enactment. We also observed a proportional rise in patients completing their referral consultation and pursuing sperm banking. During each year of our formalized FP program, more than 70% of the referred patients completed sperm cryopreservation, showing that programs such as this with adequate staffing (including fertility preservation navigators to coordinate referrals, and reproductive urologists and APPs) can be scaled to accommodate a growing patient volume.

While data on fertility preservation rates in TGNB-AMAB patients is limited, our finding are comparable with prior studies that report sperm banking utilization rates between 40 and 85% [20,21]. In a systematic review by Baram et al. that evaluated psychosocial, attitudinal, and systemic factors associated with pursuing FP, the authors found a significantly higher rate of FP utilization among patients referred for fertility consultation at specialty fertility centers than among those without these resources [22]. Overall, our results contribute to the existing literature that shows formal FP programs and referrals are effective at increasing FP completion rates among TGNB-AMAB patients.

The FP referral process, which consists of a screening telephone interview by our Fertility Preservation Program Manager followed by referral consultation to a reproductive urology MD/APP, provides minimal delay to both sperm banking and time to initiation of GAHT after banking. The delay in the initiation of hormonal therapy is often cited as a significant concern and barrier to care in TGNB-AMAB patients wishing to undergo FP [23,24]. Consequently, prioritizing FP at the expense of delaying the initiation of GAHT for gender dysphoria is a dilemma often faced by TGNB-AMAB patients.

Our study found a median time of two weeks from referral to sperm banking and of less than one month from initial referral to the initiation of GAHT. In a prior survey study by Persky et al., the authors found that less than 5% of transgender and gender-diverse youth respondents would delay GAHT by more than three months in order to undergo FP [25]. The relatively short duration from referral to the initiation of GAHT among patients undergoing FP in our study indicates that delays in the initiation of GAHT can be avoided when a formalized FP program is established.

We found no significant difference in the rate of referral completion or rate of pursuing sperm banking between pediatric/adolescent and adult patients. Pediatric/adolescent patients had a significantly lower average number of vials per sperm banking procedure, lower ejaculate volume, sperm morphology, and TMSC than adults. However, the average TMSC among pediatric patients was above the requisite clinical threshold for intrauterine insemination or other ART procedures such as in vitro fertilization [26]. These data support prior studies that show adolescents pursuing FP to have significantly lower semen parameters than adults [27].

Previous studies also show that over 70% of young adolescent TGNB-AMAB patients wished to be the sole decision-maker (without parental input) on whether to pursue FP [25]. Taken together, our findings underscore the importance of providing age-appropriate counseling for pediatric patients. They pursue FP at a similar rate to adults when allowed access to a formal FP program and can provide semen samples with adequate parameters to become biological parents in the future.

Lastly, our study found no difference in semen parameters between patients with and without prior use of GAHT. However, prior studies show conflicting results. Adeleye et al. analyzed semen parameters among 28 TGNB-AMAB patients without prior GAHT (n = 18), prior GAHT (n = 3), and current GAHT use (n = 7). In their cohort, TGNB-AMAB patients with prior and current GAHT use had significantly lower semen parameters than patients without prior GAHT, even following a median discontinuation time of 4.4 months (range: 3–6.5 months) among the cohort with prior exposure [15]. Similarly, Rodriguez-Wallberg et al. compared 161 TGNB-AMAB patients without prior GAHT with 16 patients with prior GAHT and demonstrated persistently worse bulk semen parameters among patients with prior use of GAHT, even with a range of discontinuation times between 1 and 5 months [12]. It is possible that the patients in our study with prior GAHT may have had a longer interval between cessation of GAHT and banking, leading to the normalization of semen parameters.

## 5. Limitations

Our study must be considered in the context of certain limitations. We lack information on the duration of prior GAHT, as well as the specific timing of GAHT cessation before sperm banking. The GPP is part of a tertiary academic center of excellence, and the majority of our patient population is referred to our institution by outside providers. Therefore, we lack more granular data on the patients’ prior GAHT regimen, duration of treatment, and duration of cessation prior to sperm banking. Further studies are required to determine the optimal taper regimen, if needed, and timing of GAHT cessation prior to sperm banking to potentially improve semen parameters in this population.

Second, it is possible that patients referred to the FP program may not fully represent all TGNB-AMAB individuals seeking gender-affirming care, potentially introducing selection bias in our population. Although all care is provided through the GPP with a limited set of medical providers, there may be inherent variability in counseling practices and patient decision-making regarding fertility preservation, which could affect the referral patterns and outcomes overall. This is a critically important consideration as prior studies have found that, even among patients who receive FP counseling, nearly one-quarter of patients report that the information provided was insufficient [28]. Additionally, our data lacks long-term follow-up information on reproductive outcomes. This prevents the current assessment of the success of FP in achieving the patients’ desired reproductive goals.

## 6. Conclusions

Our FP program significantly increased the number of TGNB-AMAB patients who received consultation and underwent sperm cryopreservation since its inception. The institution of a formal FP program can improve access for TGNB-AMAB patients who desire future fertility and expedite their time to sperm banking and subsequent GAHT. Establishing and expanding formalized FP programs at other institutions could play a pivotal role in enhancing the rates of FP in this patient population.

## Figures and Tables

**Table 1:** Number and percentage of TGNB-AMAB patients who were referred to FP program, completed referral, and submitted a semen sample for cryopreservation by year.

Year	Total Referrals (N)	Completed Referrals (n, %) *	Samples Provided (n, %) *
2016	5	4 (80.0)	4 (80.0)
2017	8	8 (100.0)	8 (100.0)
2018	11	9 (81.8)	8 (72.7)
2019	10	8 (80.0)	8 (80.0)
2020	15	14 (93.3)	12 (80.0)
2021	34	32 (94.1)	31 (91.2)
2022	29	24 (82.8)	23 (79.3)
2023	42	32 (76.2)	30 (71.4)
***p*-value**	0.001	0.22	0.24

* Cochran–Armitage test for trend used to determine *p*-values.

**Table 2:** Cohort demographics and clinical characteristics overall and between pediatric and adult patients.

	Overall (N = 154)	<18 Years Old (n = 71)	≥18 Years Old (n = 83)	*p*-Value (<0.05)
Age at referral (years)	20.5 ± 5.7	16.1 ± 1.6	24.3 ± 5.3	**<0.001**
Prior hormonal therapy	36 (23.4)	10 (14.1)	26 (31.3)	**0.01**
**Referral Characteristics**				
Completed referral (saw MD/APP)	131 (85.1)	61 (85.9)	70 (84.3)	0.78
Sperm banking	124 (80.5)	54 (76.1)	70 (84.3)	0.20
Number of times banking	2.1 ± 1.5	2.4 ± 1.4	1.9 ± 1.6	0.12
Vials per bank	4.4 ± 3.1	2.8 ± 2.2	5.7 ± 3.2	**<0.001**
**Semen Parameters**				
Ejaculate volume (mL)	2.7 ± 1.7	1.9 ± 1.1	3.4 ± 1.7	**<0.001**
Sperm concentration (M/mL)	36.0 ± 31.6	30.7 ± 29.5	40.1 ± 32.9	0.10
Sperm motility (%)	56.8 ± 19.0	53.9 ± 19.9	59.0 ± 18.0	0.13
Sperm morphology (%)	4.7 ± 2.9	4.0 ± 2.9	5.2 ± 2.7	**0.03**
Total motile sperm count (M)	61.4 ± 57.3	36.4 ± 41.1	80.9 ± 60.7	**<0.001**
Serum Hormonal Values				
Testosterone (ng/dL)	407.4 ± 188.6	412.3 ± 184.5	402.7 ± 194.1	0.81
FSH (mIU/mL)	3.3 ± 3.4	2.9 ± 1.6	3.6 ± 4.6	0.48

APP = advanced practice provider, FSH = follicle-stimulating hormone, MD = medical doctor.

**Table 3:** Comparison of semen parameters between patients with and without prior GAHT who completed sperm cryopreservation.

	Prior GAHT (n = 30)	No Prior GAHT (n = 94)	*p*-Value (<0.05)
Ejaculate volume (mL)	2.7 ± 1.3	2.7 ± 1.8	0.95
Sperm concentration (M/mL)	26.8 ± 23.5	38.1 ± 33.5	0.09
Sperm motility (%)	51.5 ± 20.2	58.5 ± 18.4	0.08
Sperm morphology (%)	4.2 ± 3.3	4.8 ± 2.8	0.24
Total motile sperm count (M)	51.2 ± 63.3	62.7 ± 55.4	0.34

GAHT = gender-affirming hormonal therapy.

## Data Availability

The original contributions presented in this study are included in the article. Further inquiries can be directed to the corresponding author.
